# Alcohol-Based Chlorhexidine and Potassium Sorbate Rub Strengthens the Effectiveness of Traditional Hand Scrubbing and Improves Long-Lasting Effectiveness—Evaluation of Hand Preparation Protocols According to EN 12791

**DOI:** 10.3390/antibiotics13050470

**Published:** 2024-05-20

**Authors:** Elena Herráiz Soria, Luis Alou, Carlos Martin-Villa, Ricardo Becerro-de-Bengoa-Vallejo, Marta Losa-Iglesias, David Sevillano

**Affiliations:** 1 Faculty of Health Sciences, Universidad Rey Juan Carlos, 28933 Madrid, Spain; elena.herraiz@urjc.es (E.H.S.); marta.losa@urjc.es (M.L.-I.); 2 Microbiology Area-Medicine Department, School of Medicine, Universidad Complutense de Madrid, 28040 Madrid, Spain; dsevill@ucm.es; 3 Faculty of Nursing, Physiotherapy and Podiatry, Universidad Complutense de Madrid, 28040 Madrid, Spain; podologiamartinvilla@gmail.com (C.M.-V.); ribebeva@enf.ucm.es (R.B.-d.-B.-V.)

**Keywords:** potassium sorbate, chlorhexidine, ethanol, EN 12791, surgical hand preparation

## Abstract

Despite the advantages of surgical handrub in terms of the ease of application and effectiveness, chlorhexidine (CHG)-based hand scrubbing remains the preferred method for surgical hand preparation. However, it does not systematically meet the non-inferiority requirement of the European norm (EN) 12791 with respect to n-propanol (the reference product) and does not provide the sustained efficacy expected for these long-lasting agents. Commercially available alcohol-based products have also failed to demonstrate sustained efficacy according to EN 12791. Multi-step protocols enhance the efficacy of hand scrubbing, yet their extended disinfection duration might diminish their allure for healthcare professionals. In this study, we show that hand scrubbing with CHG 4% followed by a 1 min rubbing with the novel formulation of ethanol (Et) 70%/CHG 3% plus 0.3% potassium sorbate food additive (PS) meets the non-inferiority requirement and demonstrates sustained efficacy when tested according to EN 12791. The immediate and 3 h effect of this protocol was significantly higher than that of n-propanol and the homologous disinfection protocol without PS (CHG 4% hand scrub plus Et 70%/CHG 3% rub), demonstrating that the inclusion of PS confers a notable residual effect. We speculate that this non-volatile ingredient acts synergistically with CHG. This promising combination represents an alternative method for the development of new disinfection strategies.

## 1. Introduction

Surgical site infection (SSI) is a globally recognized and preventable problem associated with high morbidity and mortality that affects 0.5–5% of patients undergoing surgery [1,2]. Although the risk of infection seems low, the large volume of surgical procedures performed annually (over 300 million worldwide) makes SSI one of the most common causes of healthcare-acquired infections and the leading cause of prolonged hospital stay [1,3]. A significant spike in SSI incidence is expected in the short term due to the increasing complexity of surgical procedures, antibiotic resistance and the population of elderly and multi-pathological patients likely to undergo surgery [3,4].

Prevention remains key to reducing SSIs. Strategies such as surgical site skin preparation, perioperative antibiotic prophylaxis and surgical hand preparation have proven to be highly effective in limiting the transmission of pathogens to the surgical site [1,4,5,6]. The World Health Organization (WHO) summary for the prevention of SSIs recommends that surgical hand preparation should be carried out by traditional hand scrubbing with water and antimicrobial soaps or by using an appropriate alcohol-based handrub [7]. No differences have been found between hand scrubbing and hand rubbing in reducing the incidence of SSI (rated as moderate quality) [5,6,7].

Soaps containing chlorhexidine digluconate (CHG), povidone-iodine (PVP), para-chloro-meta-xylenol (PCMX) or triclosan (TC) are commonly used for surgical hand scrubbing [7,8]. CHG and PVP are more effective and have a broader spectrum of activity, but CHG and, to a lesser extent, PCMX or TC prolong the inhibition of bacterial growth on the skin [7,9]. Alcohol rubs offer a viable option for surgical hand preparation, showing antibacterial efficacy comparable to or better than hand scrubbing [5,6,8]. Moreover, the rapid and profound reduction in the resident skin microbiota immediately after alcohol application delays bacterial regrowth to baseline for several hours, despite lacking the residual effects of long-acting compounds such as CHG [10].

Surgical hand scrubbing remains the standard procedure for many institutions and surgical team members, primarily due to the imperative of entering the operating theater with impeccably clean hands [8,11,12]. It should be noted that using an alcohol handrub without hand washing with plain soap and water does not guarantee the effective removal of hand contaminants. The use of alcohol-based handrubs (without hand washing) is advised as an alternative prior to transitioning to subsequent surgical procedures when the hands have no visible soiling [8,11,12]. 

In any case, when selecting antiseptics, healthcare facilities should offer products with proven efficacy according to international standards that guarantee bacterial growth inhibition under the surgical glove, since glove perforation is not an uncommon event [7,8]. 

European norm 12791:2016+A1 (EN 12791), developed by the European Committee for Standardization, describes test methods to determine whether a disinfectant or antiseptic chemical has sufficient efficacy to be used for surgical hand preparation in European countries [13]. The antibacterial efficacy on the hands of the antiseptic under test (scrubbed or rubbed) must not be inferior than that of the reference product, n-propanol at 60% (rubbed for 3 min), immediately after application and 3 h after the removal of the surgical glove. In addition, if the 3 h effect is statistically superior to that of n-propanol, the tested product is deemed to have a ‘sustained effect’ [13]. 

EN 12791 remains the most stringent standard for the approval of antiseptics for use in surgical hand preparation, despite the recent relaxation of the evaluation criteria [13,14]. This is largely due to the questionable choice of n-propanol as the reference antiseptic based on its safety profile and the lack of evidence-based studies on its potential harmfulness to humans, limiting the number of marketed formulations available for hand antisepsis [7]. Its efficacy surpasses that of ethanol (Et) or isopropanol at equivalent concentrations (*v*/*v*). At 60%, it is already more effective than the isopropanol at 70%, which is used as the reference product in the US counterpart standard, the American Society for Testing and Materials (E1115) [15,16]. 

Based on the results of in vitro tests and non-standardized clinical trials, health professionals consider medicated soaps to be effective [9,17,18,19], but the antimicrobial activity of these antiseptics differs significantly under practical conditions, such as those proposed by EN 12791 [20,21,22,23]. Medicated soaps containing PVP 10%, TC 0.5–1% or PCMX 3% do not meet the non-inferiority criteria of EN 12791 and should not be used in surgical hand scrubbing [20,21,22,23]. In this context, CHG 4% soaps have resulted in an inferior immediate effect compared to n-propanol, or non-inferiority without the sustained effect expected for this long-lasting agent when applied for 3 to 5 min [18,20,21,22,24]. The rinsing of the antiseptic after hand disinfection and the deleterious effect of scrubbing on the nail hyponychium contribute greatly to the infectivity of surgical hand scrubbing [21,22,25]. 

Interestingly, 80% *v*/*v* ethanol (Et) or 75% *v*/*v* isopropanol alcohol-based formulations recommended by the WHO for hygienic and pre-surgical hand preparation in healthcare settings where commercial products are not available, as well as n-propanol itself when applied via scrubbing rather than rubbing, also fail to meet the criteria outlined in this standard [7,26], underscoring the complexity in selecting antiseptics that satisfy the effectiveness requirements of the European standard. 

For these reasons, several studies have suggested the use of multi-stage disinfection protocols to ensure that traditional hand scrubbing meets the requirements of EN 12791 [21,22,23]. Protocols that supplemented traditional hand scrubbing with a rub using CHG or PVP aqueous solutions or alcohol-based solutions met the non-inferiority requirement [21,22,23], but only traditional hand scrubbing with CHG 4% followed by rubbing with aqueous CHG 5% showed sustained effects at the cost of a marked increase in the duration of the process [21]. Despite the improved effectiveness, a balance between the effectiveness and duration of the disinfection process is needed to promote adherence to the disinfection procedure [22,23]. 

The alcohol-based CHG and potassium sorbate (PS) solution, Et70% (*v*/*v*), CHG3% (*v*/*v*) and PS 0.3% (*v*/*v*) (Et/CHG/PS), named Sorbectol^®^ [27], is a novel antiseptic product formulated to provide the sustained inhibition of hand microbiota beyond that achieved by alcohols. Although several alcohol-based hand sanitizers with CHG have been licensed, there is strong controversy over the need to include this long-lasting ingredient in hydroalcoholic formulations, because the immediate and profound reduction in bacterial load produced by alcohols in clinical practice already extends the inhibition of bacterial regrowth on the hands for several hours [10,11,12,13,14,15,16,17,18,19,20,21,22,23,24,25,26,27,28]. In these circumstances, it is recommended not to include non-volatile agents such as CHG in alcohol-based formulations in order to reduce the risk of skin irritation [29].

The new alcohol-based formulation, alongside the typical WHO-recommended Et concentration for hand hygiene (70%), includes CHG 3% and a safe food additive, PS, which, like CHG, has residual in vitro effects [30]. The intrinsic activity of PS is poor, but a recent study revealed that its antimicrobial efficacy substantially increases in bacteria subjected to hyperosmotic stress, outperforming CHG 0.2% [31]. We hypothesize that the osmotic stress induced by CHG may enhance the action of PS, resulting in a prolongation of the antimicrobial effect.

Traditional hand scrubbing is considered the gold standard technique by many surgical teams. Given the lack of efficacy of the procedure when evaluated according to the EN standard, in this study, we set out to evaluate the efficacy of a two-step surgical hand preparation protocol that supplements traditional CHG hand scrubbing with a short-duration handrub using the new alcohol-based CHG and PS solution (Et/CHG/PS). This study is the first to bring this novel formulation to clinical practice.

## 2. Results

The study enrolled 24 out of 30 potential participants (Table S1, Figure S1). All 24 volunteers successfully completed the three experimental antisepsis arms. The quality of the test results was found to be acceptable and could therefore be used for the evaluation of the products under test according to the effectiveness criteria of EN 12791 (Appendix A).

The mean bacterial load before antisepsis (immediate or 3 h pre-values) was very similar in the three disinfection protocols, ranging from 3.85 to 4.15 log10 (*p* > 0.05 for all comparisons), as shown in Table 1. As Table 1 shows, the three procedures, P1, P2 and RP, significantly reduced (*p* = 0.0001) the initial bacterial load on the hands immediately after antisepsis (immediate post-values) and under gloves 3 h later (3 h post-values). P1 gave the highest immediate reduction in bacterial load on the hands and inhibited bacterial regrowth under the glove during the 3 h test. The immediate and 3 h bacterial load reduction was not significantly different (*p* = 0.683). In contrast, with P2 and RP, the reduction in bacterial load at 3 h was significantly lower (*p* ≤ 0.0004) than the immediate reduction. 

Table 2 shows the difference in the antimicrobial activity of the RP procedure compared to P1 and P2. The P1 protocol caused a significantly higher immediate (*p* < 0.0001; one-tailed) and 3 h (*p* < 0.0001; one-tailed) effect than the RP protocol, fulfilling the non-inferiority and sustained effect requirements proposed by EN 12791. The P2 protocol, although significantly superior to RP at 3 h (*p* = 0.045 one-tailed), met the requirement of non-inferiority but without the prolonged effect according to EN 12791.

The addition of PS 0.3% to the alcohol-based CHG solution significantly increased the antimicrobial activity of protocol P1 compared to protocol P2 immediately on the hands (*p* = 0.0002; two-tailed) and under the glove 3 h after antiseptic application (*p* < 0.0001; two-tailed).

None of the volunteers experienced any adverse reactions after the application of the disinfection protocols.

## 3. Discussion

Despite the advantages in terms of the ease of application, reduced time consumption, low-level skin irritation and better dermal tolerance of the alcohol-based product, hand scrubbing with a sponge or brush soaked with CHG or PVP is still preferred by many surgical teams due to issues related to safety, efficacy and effectiveness [8,32]. With similar efficacy in terms of reducing SSIs and at no higher cost, traditional hand scrubbing is a simple technique, less prone to application error, that ensures the removal of visible soiling and bacterial spores from the hands prior to entering the operating theater for the first time, in the same way as hand washing with plain soap followed by rubbing with an alcohol-based solution [8].

However, the priority of surgical hand preparation should be based on the effectiveness of the process rather than on any personal preference and, in this sense, the efficacy of hand scrubbing is uncertain when assessed against EN 12791 [20,21,22,23]. Reapplication of the scrub or a combination of the scrub with an alcohol-based rub to improve the effectiveness of surgical hand scrubbing, prolonging the inhibition of bacterial growth on the skin for hours, is supported by multiple efficacy studies not guided by EN 12791 [9,17,19]. However, as far as we are aware, only three multi-step protocols met the non-inferiority requirement of EN 12791 [21,22,23], and only the traditional hand scrub with CHG 4% (5 min) supplemented with aqueous CHG 5% rub (3 mL for 3 min and 2–3 min of drying time) proposed by Herruzo was able to meet the sustained effect requirement of EN 12791 [21,22,23,24,25,26,27,28,29,30,31,32,33,34,35]. In agreement with the unstandardized efficacy studies [17,33,34], protocols complemented with PVP [21] or Et [23] rubs were associated with rapid bacterial regrowth. Importantly, in all three trials, the residual activity of the antiseptics was neutralized in the sampling fluid [35]. Neutralization is an essential technical step to accurately determine the number of surviving bacteria on the hand, as antiseptics, and especially non-volatile agents, can remain active in culture media. Without proper neutralization, the clinical efficacy of antiseptics is often overestimated [29].

In this study, we showed that at least one of the two tested surgical hand preparation protocols met the sustained effect requirement of EN 12791, with a marginal extension of the disinfection protocol compared to the protocol evaluated by Herruzo [21]. The application of both alcohol-based solutions (Et/CHG with or without potassium sorbate) after traditional hand scrubbing with CHG 4% took just 1 min and hand drying before gloving was almost immediate. Both protocols tested passed the non-inferiority requirements of EN 12791, and both reduced the bacterial load at 3 h to a greater extent than n-propanol. However, only the P1 protocol, which included the new formulation of Et/CHG/PS, inhibited bacterial growth under the glove to the extent that it can be claimed to have a sustained effect according to EN 12791. Toxicity and neutralization tests for the mixture of neutralizing agents were validated according to UNE 13729 and were within the recommended limits of the standard, despite the higher variability seen for the Et/CHG/PS formulation (Table S2). The impact of using neutralizing agents in the sampling liquid was only limited for this formulation, as most valid counts were obtained from undiluted samples. 

Our experimental design did not include a separate arm for traditional CHG hand scrubbing, and therefore we do not know the exact contribution of the hydroalcoholic formulations to the effect. However, we speculate that the activity of P1 and P2 was essentially due to the alcohol-based rubs, as our group previously tested the effectiveness of surgical hand scrubbing with CHG 4% for 5 min using the same scrubbing method and, far from meeting the efficacy criteria of EN 12791, the procedure caused a paradoxical over-colonization of the hands immediately after the application of the antiseptic [22]. Moreover, here, unlike in our previous study [22], the residual activity of the antiseptics was neutralized. In any case, the contribution of potassium sorbate to the effect of protocol P1 is not in doubt, as this additive was the differential ingredient of both protocols, allowing for a direct comparison of their efficacy [10]. Thus, the significant superiority of the P1 protocol over the P2 protocol in terms of the immediate and 3 h effects can be attributed to the inclusion of PS in the alcohol-based solution.

The use of long-lasting agents in alcohol formulations remains controversial. Efficacy studies with commercially available alcohol-based hand sanitizers support that optimized, well-formulated alcohol-only solutions offer superior performance to alcohol-based solutions containing long-acting agents, including the prolonged inhibition of bacterial effect [10,28,34,36]. However, the variability in the alcohol composition of formulations is an important confounding factor in accurately assessing the residual effect of long-lasting agents (e.g., Softa-Man^®^; Et 45% and n-propanol 18% vs. 3MTM AvagardTM; CHG 1% and Et 61% vs. n-propanol 60% (*v*/*v*)) [28,34,36]. In any case, there is currently no conclusive evidence to support a sustained effect according to EN 12791 for any alcohol-based solution, with or without long-lasting non-volatile ingredients [10]. 

In our study, the application of Et/CHG failed to provide P2 with a sustained effect as the application of Et 70% (for 3 min) after surgical hand scrubbing with TC did previously in the study by Sante et al. [23], highlighting the residual effect that PS brings to the hydroalcoholic formulation, conferring a sustained effect to traditional hand scrubbing with CHG 4%.

The mechanism of action of the Et/CHG/PS formulation is currently unknown. However, we speculate that the synergistic action of both non-volatile ingredients, CHG and PS, contributed to enhancing the antibacterial effect rather than merely an additive antimicrobial effect due to the incorporation of PS. Potassium sorbate (E202) is a safe and well-tolerated agent (acceptable daily intake up to 25 mg/kg body weight per day) used for technological purposes in food preparation, storage and preservation [30,37]. From a microbiological viewpoint, it is bacteriostatic, with limited intrinsic activity and a reduced spectrum of action compared to classical antiseptics [30]. 

At a physiological pH, PS dissociates into potassium and sorbate ions, and, to a lesser extent, as sorbic acid [38,39]. As a weak hydrophobic acid, sorbic acid diffuses across the membrane into the cell, dissociating in a new equilibrium with sorbate anions and protonating the cytosol. The extrusion of protons at the expense of ATP to maintain pH homeostasis reduces the proton gradient, increasing the energy demand and decreasing the rate of glucose uptake. This leads to nutrient limitation. In addition, sorbate ions have been shown to alter the membrane and interfere with membrane proteins and affect cytosolic enzymes through changes in osmolarity [39,40]. 

However, van der Walls et al. showed that hyperosmotic stress significantly enhanced the antimicrobial effect of PS [31], hypothesized to be due to the increased intracellular uptake of PS ions, to restore osmotic balance and sorbic acid.

In our opinion, the mechanism of action of the CHG/PS combination is reminiscent of that described by van der Walls [31], being a result of hyperosmotic stress caused by the interaction of chlorhexidine with the microbial cell membrane. Chlorhexidine at low concentrations disturbs cell membrane permeability, causing dysfunction in osmoregulation and the metabolic efficiency of membrane enzymes, and resulting in the leakage of potassium ions and protons from the microbial cell, alongside the inhibition of respiratory activity and the transport of dissolved substances [41]. In response to this hyperosmotic stress, the entry of sorbate ions further destabilizes cell membranes and promotes the intracellular penetration of chlorhexidine. Thus, the combination of hyperosmotic stress and the direct action of chlorhexidine, sorbate ions and sorbic acid on cell membranes and the cell metabolism triggers a cascade of events leading to the death of the microorganism.

The substantivity of chlorhexidine is critical to understanding the prolongation of action under the glove. We believe that the concentrations required are low, given that the hands were rinsed with water after washing with CHG 4%.

In conclusion, employing two-step disinfection protocols for surgical hand preparation, which involve a brief rub with an optimized alcohol-based solution as the new alcohol-based chlorhexidine and potassium sorbate formulation, could prove to be a valuable strategy for maintaining the effectiveness of traditional hand scrubbing, as outlined by the rigorous EN 12791. This approach appears promising, provided that the current acceptance and adherence to traditional hand scrubbing among surgical teams persist. Despite the positive results, traditional hand scrubbing should gradually give way to alcohol-based rubs, which are well-documented to be effective and support shorter surgical hand preparation protocols. Our study indicates that long-lasting compounds can improve the sustained effect of alcohol-based solutions. These encouraging findings justify additional clinical trials to evaluate whether the alcohol-based chlorhexidine and potassium sorbate solution meets the non-inferiority and sustained effect criteria outlined in EN 12791 when used following preparative hand washing without traditional hand scrubbing.

## 4. Materials and Methods

### 4.1. Study Design and Participants

A randomized controlled trial with a Latin-square crossover design was conducted from July 2020 to October 2020 to test a two-step surgical hand preparation procedure consisting in standard surgical hand scrubbing with CHG 4% followed by hand rubbing with the sorbate potassium hydroalcoholic formulation Et/CHG/PS (P1) or by hand rubbing with the Et 70%/CHG 3% (Et/CHG) base solution (P2) with respect to n-propanol handrub (RP), according to the European standard EN-12791 [13]. 

The Ethics Committee for Clinical Research of the Hospital Universitario Clínico San Carlos, Madrid, approved the trial; ID 20/389-EC (ClinicalTrials.gov; NCT04454619). 

Volunteers assessed for eligibility (*n* = 30) were instructed on the EN 12791 test procedure and the technique for the application of surgical hand antisepsis and glove-wearing recommended by the WHO [7]. The inclusion criteria defined in the EN 12791 document were adopted, with special emphasis on avoiding any contact with topical antiseptics, including creams with biocidal ingredients, for 3 days prior to testing and oral antibiotics for at least 10 days prior to testing. Enrolled participants (Table S1) were randomly divided into three groups of the same size (Figure S1) to receive RP, P1 and P2 in parallel in a first run. The test was repeated in a second and third run, changing the antisepsis roles for each group. A washout period of at least 2 weeks between experimental runs allowed for the reconstitution of skin microbiota. All participants had completed the three antiseptic procedures at the end of the trial.

### 4.2. Procedure

After the preparatory hand wash using a diluted soft soap (5 mL for 1 min), all fingertips were rubbed for 1 min on the base of two Petri dishes (one per hand) containing 10 mL of sampling fluid (tryptone soy broth—TSB; Becton Dickinson, Franklin Lakes, USA) to assess the bacterial load on the hands before antisepsis (pre-values). Sample dilutions (1:10–1:100) on TSB were spread (0.1 mL) on the surface of tryptone soy agar (TSA, Becton Dickinson, Franklin Lakes, USA) plates and incubated for 24 h prior to colony-forming unit (CFU) counting. Results were expressed as the decimal logarithm (Log) of CFUs/mL. 

Next, surgical hand antisepsis protocols were performed. Volunteers assigned to the RP group rubbed their hands with n-propanol (60% *v*/*v*), applying as many 3 mL portions (at least 3 portions) as necessary to keep the hands moist for 3 min. After the evaporation of the alcohol (<20 s), the hands were gloved. Volunteers in the P1 and P2 groups used a sterile surgical brush/sponge impregnated with 20 mL CHG4% (Euronda, Vicenza, Italy) for 5 min [22] to scrub the entire hand, including the nails, thoroughly for 3 min and the forearms for 2 min. The hands were rinsed with running water and dried with sterile towels (DIRRA, Vidiana, Italy). Then, the hands were rubbed with the Et/CHG/PS (group P1 participants) or Et70/CHG (group P2 participants) formulations, applying a sufficient volume of antiseptic to keep the hands moist for 1 min (approximately 3 mL). Immediately after drying (<20 s), the hands were gloved. 

The bacterial load after the antisepsis procedures (post-values) were determined using a split-hands model. One hand was assigned to assess the bacterial load immediately after treatment (immediate post-values) and the other hand was assigned to assess the bacterial load 3 h later, keeping the hand gloved in the meantime (3 h post-values). According to the EN 12791 standard, the role assigned to each hand in the first run was swapped in the subsequent runs (Figure S1). The sampling method described for the pre-values was used for the post-values, but neutralizing agents were included in the sampling fluid. In addition, undiluted samples (1 mL) were plated on TSA. The neutralizing agent was the mixture of 3% Tween 80 (PanReac-AppliChem, Barcelona, Spain), 3% saponin (Panreac-AppliChem) and 0.3% egg lecithin (PanReac-AppliChem) as recommended by EN 13727 [42] for the neutralization of biguanides and alcohol compounds. The toxicity of the neutralizing mixture and the neutralizer effect were evaluated following the test method proposed by EN 13727 (Table S2). 

### 4.3. Outcome Measures

The primary outcomes measures were bacterial reductions (in Lg) obtained immediately (LogR-I) and 3 h (LogR-3h) after surgical hand antisepsis. The LogR-I was calculated as the Log pre-values—Log immediate post-values obtained on the same hand for each experimental protocol, while the LogR-3h was calculated as the Log pre-values—Log 3 h post-values obtained on the other hand. Primary outcome measures were used to evaluate the non-inferiority and sustained effect criteria proposed by EN 12791 for the tested antiseptic protocols, P1 and P2 vs. RP.

The secondary outcome measures were the Log pre-values and Log post-values (immediate and 3 h) established for the different experimental protocols.

### 4.4. Verification of the Methodology

The results of the test procedure were inspected to ensure that they met the acceptance requirements of EN 12791, which were as follows: (i) include a complete test result for at least 23 volunteers; (ii) overall means of Log pre-values for RP, P1 and P2 of at least 3.5; (iii) an absolute difference in the mean differences between the Log reductions (LogR-I or LogR-3h) obtained for the P1 and P2 groups when tested before and after RP (test of sequence of effects), <2.

Compliance with these quality requirements enabled the evaluation of the effectiveness of the products under test according to EN 12791 (Appendix A).

### 4.5. Statistical Analysis

The nonparametric Wilcoxon matched-pairs signed-rank test (*p*-value = 0.05; two-tailed test) was used for pre- vs. post-values or LogR-I vs. LogR-3h comparisons. The Friedman nonparametric test for related samples (*p*-value = 0.05; two-tailed test) was used for comparison between the three independent groups. The test was set at *p* = 0.025 (one-tailed) to validate the non-inferiority of the immediate and 3 h effect of P1 or P2 versus RP, and at *p* = 0.01 (one-tailed) to validate the sustained effect criterion. The one-tailed *p*-value was calculated as the *p*-value/2 for the RP vs. P1 or P2 median difference. All of the analyses were performed using the GraphPad Prism software v 8.01 (San Diego, CA, USA).

## Figures and Tables

**Table 1:** Bacterial counts for pre-values and post-values and immediate (LogR-I) and 3 h (LogR-3h) bacterial reduction (in Log CFU/mL) for the RP and surgical hand procedures P1 and P2.

Surgical Hand Preparation Protocol	Immediate Test			3 h Test			
	Pre-Value	Post-Value	LogR-I	Pre-Value	Post-Value	LogR-3h	LogR-I vs. LogR-3h
**RP**	3.85 ± 0.38 (3.89)	1.79 ± 1.01 * (1.78)	2.06 ± 1.02 (1.99)	3.96 ± 0.45 (3.63)	3.01 ± 0.57 * (3.11)	0.94 ± 0.74 (0.93)	<0.0004
**P1**	4.02 ± 0.53 (4.02)	0.58 ± 0.78 * (0.24)	3.43 ± 0.65 (3.50)	4.15 ± 0.47 (4.20)	0.67 ± 0.81 * (0.15)	3.48 ± 0.86 (3.63)	0.6381
**P2**	3.98 ± 0.70 (3.98)	1.61 ± 0.66 * (1.30)	2.36 ± 0.76 (2.39)	4.08 ± 0.64 (4.13)	2.64 ± 0.78 * (2.55)	1.44 ± 0.85 (1.59)	<0.0001

RP, n-propanol 60%. P1, CHG 4% (hand scrub 5 min) followed by the Et/CHG/PS solution (handrub, 1 min). P2, CHG 4% (hand scrub, 5 min) followed by the Et/CHG solution (handrub, 1 min). Data are expressed as the mean ± standard deviation (median). LogR-I for P1 and immediate pre-values, post-values, LogR-I and LogR-3h for P2 did not fit a normal distribution (Shapiro–Wilk test; *p* < 0.05). * *p*-value < 0.001 with a 95% confidence interval for pre-values vs. post-values using the Wilcoxon matched-pairs signed-rank test.

**Table 2:** Mean/median of the differences in immediate (LogR-I) and 3 h effects (LgR-3h) between the reference product and surgical hand preparation protocols P1 or P2 and non-inferiority/sustained evaluation criteria according to EN 12791.

Surgical Hand Preparation Protocols	Immediate Effect			3 h Effect			
	Mean (Median) of the Differences	*p*	Fulfils Non-Inferiority Criterion *	Mean (Median) of the Differences	*p*	Fulfils Non-Inferiority Criterion *	Fulfils the Sustained Effect Criterion **
**RP-P1**	−1.37 (−1.42)	0.0001	Yes	−2.54 (−2.325)	<0.0001	Yes	Yes
**RP-P2**	−0.30 (−0.025)	0.5	Yes	−0.50 (−0.16)	0.0455	Yes	No

RP, n-propanol 60%. P1, CHG-4% (hand scrub) followed by the Et/CHG/PS solution (handrub). P2; CHG 4% (hand scrub) followed by the Et/CHG solution (handrub). * *p*-value (one-tailed) for the median of RP-P difference using the Friedman test. ** *p*-value was set at 0.025 for the assessment of the non-inferiority criterion for the immediate and 3 h effect, and at <0.01 for validations of the sustained effect criterion.

## Data Availability

The raw data supporting the conclusions of this article will be made available by the authors on request.

## Supplementary Materials

The following supporting information can be downloaded at: https://www.mdpi.com/article/10.3390/antibiotics13050470/s1, Table S1: Sociodemographic characteristics of the enrolled participants; Figure S1: Crossover clinical trial flow chart; Table S2. Validation test for the neutralizing agent according to EN 13727.

## Appendix A

Acceptance criteria for the test results.

EN 12791 comprises multiple essential criteria that need to be met for the test procedure’s outcomes to be deemed acceptable for the evaluation of the effectiveness of the products under test.

- A complete set of results for at least 23 volunteers.
  A total of 24 complete sets for the immediate effect and a total of 24 sets for the 3 h effect were obtained.
- An overall means for Log pre-values ≥3.5 CFU/mL.
  - Overall means of Log pre-values RP (immediate/3 h pre-values): 3.85/3.96;
  - Overall means of Log pre-values PP1 (immediate/3 h pre-values): 4.02/4.15;
  - Overall means of Log pre-values PP2 (immediate/3 h pre-values): 3.98/4.08.
- The test sequence shall be ≤2. The test sequence was calculated as the absolute difference in the mean differences between the LogR-I or LogR-3 hour for RP and P1 or P2 of the subgroups where RP was used before (RP->P1 or RP->P2) and after P1 or P2 (P1->RP or P2->RP), according to the expression |RP->P1 or RP->P2| − |P1->RP or P2->RP|.
  PP1
  - Between the groups RP->P1 and P1->RP for LogR-I = 0.92 (RP->PP1; −0.76, n = 8. PP1->RP; −1.68, n = 16);
  - Between the groups RP->P1 and P1->RP for LogR-3h = 0.63 (RP->PP1; −2.12, n = 8. PP1->RP; −2.75, n = 16).
  - PP2
  - Between the groups RP->P2 and P2->RP for LogR-I = 0.93 (RP->PP1; −0.14, n = 16. PP1->RP; −1.07, n = 8);
  - Between the groups RP->P2 and P2->RP LogR-3h = 0.25 (RP->PP1; −0.58, n = 16. PP1->RP; −0.33, n = 8).

Therefore, all acceptance criteria were fulfilled.
