# Supplementary material for: Alcohol-Based Chlorhexidine and Potassium Sorbate Rub Strengthens the Effectiveness of Traditional Hand Scrubbing and Improves Long-Lasting Effectiveness—Evaluation of Hand Preparation Protocols According to EN 12791
Source: Antibiotics (Basel). 2024 May 20;13(5):470. doi: 10.3390/antibiotics13050470 (PMC11117658; doi:10.3390/antibiotics13050470)
Supplement: Supplementary file 1 [file antibiotics-13-00470-s001.zip › antibiotics-3011939-supplementary.pdf]

antibiotics

MDPI

Supplementary Material

Table S1. Sociodemographic characteristics of the enrolled participants.

Variables	Total (n=24)		Male (n=10)		Female (n=14)		*p* value
**Descriptive data**	**Mean±SD (95%CI)**	**Median (IR)**	**Mean±SD (95%CI)**	**Median (IR)**	**Mean±SD (95%CI)**	**Median (IR)**	
Age (years)	29.70±8.56 (26.27-33.13)	27.59 (7,50)	34.20±10.98 (27.30-41.00)	28.50 (15.75)	29.71±7.37 (25.46-33.97)	25.50 (7.50)	.026[a]
Weight (kg)	68.33±12.70 (63.25-73.41)	65.50 (19.50)	79.60±8.72 (74.19-85.00)	78-00 (14.75)	67.29±12.18 (60.25-74.32)	60.00 (12.75)	<.001[b]
Height (m)	1.62±0.02 (1.60-1.64)	1.61 (0.05)	1.77±0.06 (1.72-1.82)	1.80 (0.13)	1.62±0.02 (1.66-1.73)	1.61 (0.05)	<.001[a]
BMI	23.22±2.58 (22.18-24.25)	23.67 (3.44)	25.12±1.65 (24.10-26.15)	25.36 (2.44)	23.08±3.06 (21.30-24.85)	22.30 (5.10)	.001[b]

Abbreviations: BMI, body mass index; CI, confidence interval; SD, standard deviation; Kg, kilograms; M, meters. [a]*p* value for U Mann Whitney and [b]*p* value for unpaired t-test according to Shapiro-Wilk test. Statistical significance for a *p* value < .05 with a 95% confidence interval.

The Shapiro-Wilk test was used to check if variables fit a normal distribution. For parametric and nonparametric independent variables, the independent T-test and the Mann Whitney U-test was used, respectively.

The table shows that all volunteers were aged 18 years or older (21-38 years), fulfilling the only socio-demographic criterion recommended by the EN 12791. The heterogeneity of the population in terms of gender, height or weight does not affect the results due to the design of the study, in which each volunteer serves as his/her own control.

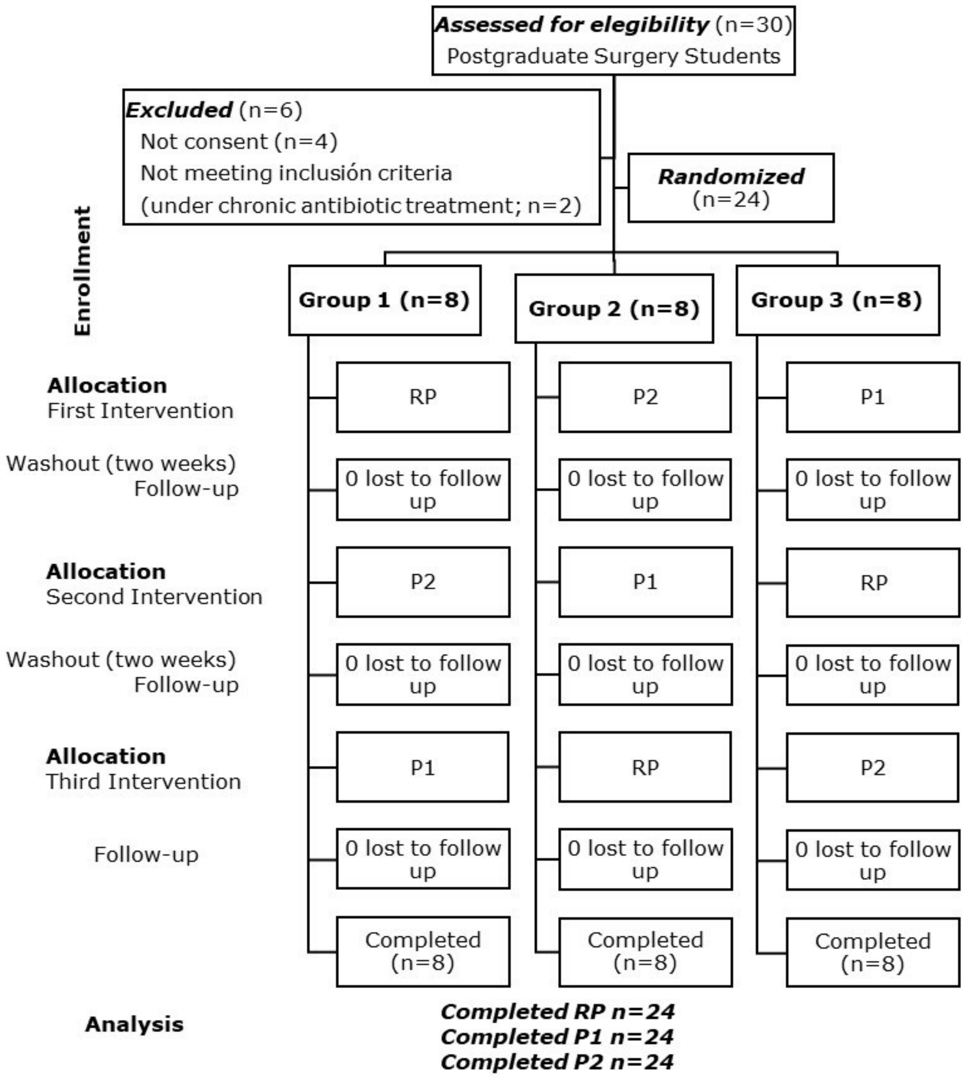

Figure S1. Cross-over clinical trial flow chart. RP; n-propanol 60%. P1; CHG 4% (hand scrub) followed by Et/CHG/PS solution (handrub). P2; CHG 4% (handscrub) followed by Et/CHG solution (handrub).

Briefly, participants were randomly divided into the three groups using Excel program (Office 365, Microsoft, "RANDBETWEEN" function). Allocations were concealed with sealed, numbered, tamperproof, opaque envelopes that were opened only after participants consented the enrollment.

In each group, half of the volunteers were randomly assigned to use their left hand for immediate post-value sampling and their right hand for 3-hour post-value sampling during the first run. The remaining participants followed the opposite protocol. In the next interventions, participants switched hand roles. By the end of the three interventions, half of the immediate and 3-hour post-value samples were obtained from the left hand, and half from the right hand.

Validation of neutralising agents

The toxicity and effectiveness of the neutralizer was evaluated following the test method proposed by EN 13727, using *Staphylococcus aureus* ATCC 6538 and *Staphylococcus epidermidis* ATCC 35984 as test microorganisms.

Briefly, standardised solutions of approx. 1x10^5 CFU/ml or 1x10^3 CFU/ml of each microorganism, were prepared in sterile saline from a fresh subculture. To validate the absence of neutraliser toxicity, 1 ml of the standardised 1x10^5 bacterial solution was added to 9 ml of the neutralising mixture (in saline). The resulting solution was serially diluted twice yielding a final bacterial inoculum of approximately 1x10^2 (N_0). After 30 minutes exposure, the number of CFU/ml was determined.

To evaluate the effectiveness of neutralisation, 1-ml of antiseptic was added to 8-ml of the neutralising mixture and after 5 min of contact, a standardised microbial suspension of 1x10^3 CFU/ml (N_0=final inoculum) was added. After 30 min of exposure, the number of CFU/ml was determined. As viability controls, microorganisms adjusted to N_0 were maintained for 30 min in saline. All tests were performed in three occasions.

Validation tests were acceptable if the bacterial count was equal to or greater than x0.5 N_0 (Log change of 0.3 CFU/ml for bacterial count after and before -N_0- exposure).

N_0 ranged between 1.87-2.16 Log CFU/ml for microorganisms tested. The bacterial viability and toxicity and effectiveness of the neutralizer are shown in table S2.

Table S2. Validation test for the neutralizing agent according to EN 13727.

	**Log change for bacterial count at 30 min vs N_0**					
	**Control**	**Neutralizer**	**n-propanol 60%**	**CHG 4%**	Et/CHG	Et/CHG/PS
S. epidermidis ATCC 35984	0.02±0.01	-0.01±0.01	0.03±0.01	0.07±0.02	0.05±0.04	0.10±0.04
S. aureus ATCC 6538	0.01±0.03	-0.02±0.00	0.02±0.01	0.05±0.02	0.04±0.00	0.06±0.04

Mean±standard deviation is expressed. Et/CHG; Et 70% /CHG 3%. Et/CHG/PS; Et 70%/CHG 3%/PS 0.3%.

The test results were found to be within the acceptance range proposed by EN 13727. Therefore, the neutralisation step was valid.
